# 2-{[2,8-Bis(tri­fluoro­meth­yl)quinolin-4-yl](hy­droxy)meth­yl}piperidin-1-ium tri­chloro­acetate: crystal structure and Hirshfeld surface analysis

**DOI:** 10.1107/S2056989018016389

**Published:** 2018-11-22

**Authors:** James L. Wardell, Mukesh M. Jotani, Edward R. T. Tiekink

**Affiliations:** a Fundaçaö Oswaldo Cruz, Instituto de Tecnologia em Fármacos-Far Manguinhos, 21041-250 Rio de Janeiro, RJ, Brazil; b Department of Physics, Bhavan’s Sheth R. A. College of Science, Ahmedabad, Gujarat 380001, India; c Research Centre for Crystalline Materials, School of Science and Technology, Sunway University, 47500 Bandar Sunway, Selangor Darul Ehsan, Malaysia

**Keywords:** crystal structure, mefloquine, salt, hydrogen-bonding, Hirshfeld surface analysis

## Abstract

The asymmetric unit of the centrosymmetric title salt, C_17_H_17_F_6_N_2_O^+^·C_2_Cl_3_O_2_^−^, comprises a single ion-pair. The hy­droxy-O and ammonium-N atoms lie to the same side of the cation, a disposition maintained by a charge-assisted ammonium-N—H⋯O(hy­droxy) hydrogen bond [the O_h_—C_m_—C_m_–N_a_ (h = hy­droxy, m = methine, a = ammonium) torsion angle is 58.90 (19)°]. The piperidin-1-ium group is approximately perpendicular to the quinolinyl residue [C_q_—C_m_—C_m_–N_a_ (q = quinolin­yl) is −178.90 (15)°] so that the cation, to a first approximation, has the shape of the letter *L*. The most prominent feature of the supra­molecular association in the crystal is the formation of chains along the *a*-axis direction, being stabilized by charge-assisted hydrogen-bonds. Thus, ammonium-N^+^—H⋯O^−^(carboxyl­ate) hydrogen bonds are formed whereby two ammonium cations bridge a pair of carboxyl­ate-O atoms, leading to eight-membered {⋯O⋯HNH}_2_ synthons. The resulting four-ion aggregates are linked into the supra­molecular chain via charge-assisted hydroxyl-O—H⋯O^−^(carboxyl­ate) hydrogen bonds. The connections between the chains, leading to a three-dimensional architecture, are of the type C—*X*⋯π, for *X* = Cl and F. The analysis of the calculated Hirshfeld surface points to the importance of *X*⋯H contacts to the surface (*X* = F, 25.4% and *X* = Cl, 19.7%) along with a significant contribution from O⋯H hydrogen-bonds (10.2%). Conversely, H⋯H contacts, at 12.4%, make a relatively small contribution to the surface.

## Abstract 2

The mefloquinium cation in the title salt is l-shaped as the piperidin-1-ium group is nearly orthogonal to the quinolinyl residue. Supra­molecular chains arise in the crystal as a result of charge-assisted O—H^⋯^O and N—H⋯O hydrogen-bonding.

## Chemical context   

Kryptoracemic behaviour is an inter­esting but rare phenomenon whereby enanti­omeric mol­ecules crystallize in one of the 65 Sohncke space groups. Sohncke space groups lack an inversion centre, a rotatory inversion axis, a glide plane or a mirror plane, implying *Z*′ would usually be greater than 1 (unless the mol­ecule lies on a rotation axis) and in which enanti­omeric mol­ecules, when present, are related by a non-crystallographic symmetry, *e.g*. a non-crystallographic centre of inversion. Reviews of this phenomenon have appeared for organic compounds (Fábián & Brock, 2010 ▸) and for coordination complexes (Bernal & Watkins, 2015 ▸). For organic mol­ecules, kryptoracemic behaviour is uncommon and is found in only 0.1% of structures (Fábián & Brock, 2010 ▸). It is therefore of inter­est that pharmacologically relevant (Gonçalves *et al.*, 2012 ▸) mefloquine/derivatives, for which there are about 30 structures included in the Cambridge Structural Database (Groom *et al.*, 2016 ▸), present two examples of krypto­racemates (Jotani *et al.*, 2016 ▸; Wardell, Wardell *et al.*, 2016 ▸). In order to investigate reasons for this seemingly high propensity towards kryptoracemic behaviour in mefloquine derivatives, crystallographic studies of different mefloquinium salts have subsequently been performed (Wardell *et al.*, 2018 ▸) and in a continuation of these, herein the crystal and mol­ecular structures of the title salt, (I), isolated from the 1:1 crystallization of racemic mefloquine and tri­chloro­acetic acid are described. This is complemented by an analysis of its calculated Hirshfeld surface.
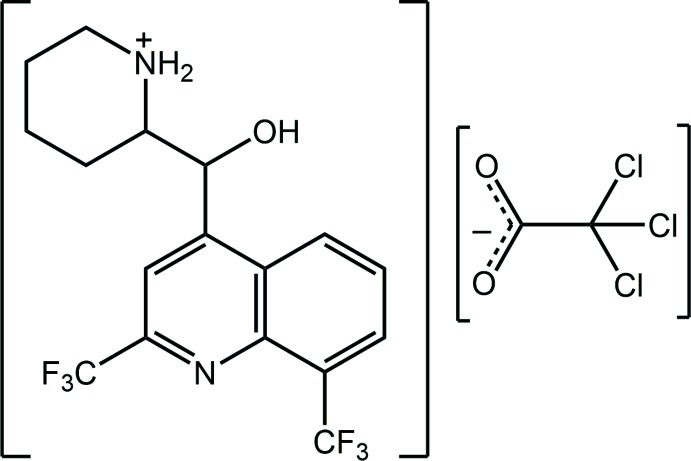


## Structural commentary   

**Figure 1:**
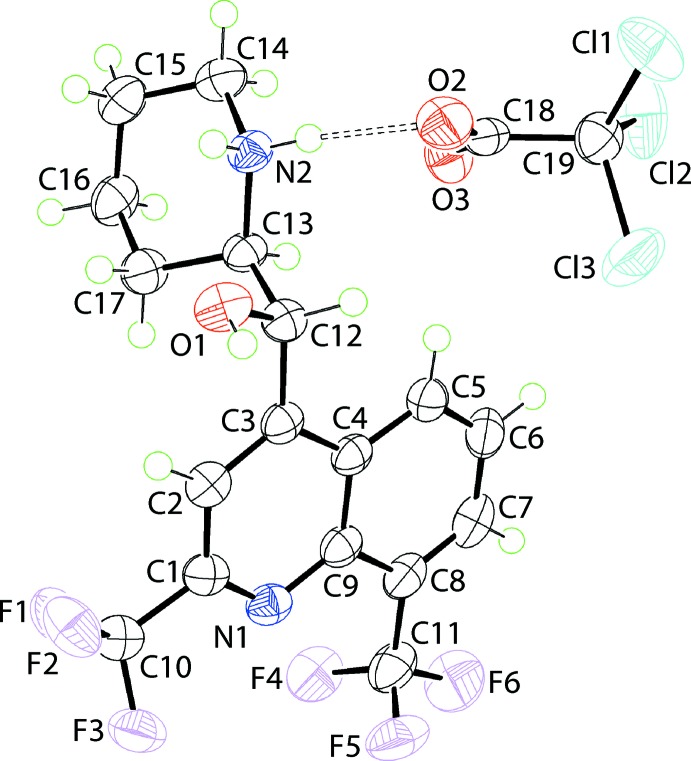
The mol­ecular structures of the two ions comprising the asymmetric unit of (I) showing the atom-labelling scheme and displacement ellipsoids at the 70% probability level. The dashed line signifies an N—H⋯O hydrogen bond.

The two ions comprising the asymmetric unit of salt (I) are shown in Fig. 1 ▸. The crystal of (I) is racemic. Each cation contains two chiral centres and the illustrated cation in the arbitrarily chosen asymmetric unit is *S* at C12 and *R* at C13, *i.e*. conforming to the [(−)-*erythro*-mefloquinium] isomer. That protonation from the carb­oxy­lic acid to the base occurred during co-crystallization is readily seen in the equivalence of the C18 O2, O3 bond lengths, *i.e*. 1.238 (3) and 1.245 (3) Å, respectively. The formation of the piperidin-1-ium cation is supported by the pattern of hydrogen bonding involving the ammonium-N—H H atoms. Indeed, an intra­molecular ammonium-N^+^—H⋯O(hy­droxy) hydrogen bond is formed ensuring the hydroxyl-O1 and ammonium-N2 atoms are orientated to the same side of the cation with the O1—C12—C13—N2 torsion angle of 58.90 (19)° angle indicating a + syn-clinal relationship. The r.m.s. deviation for the 10 atoms comprising the quinolinyl residue is 0.0147 Å, with the hy­droxy-O1 [−0.299 (3) Å] and ammonium-N2 [1.490 (4) Å] atoms lying to either side of the plane. The dihedral angle of 74.00 (5)° formed between the fused ring system and the best plane through the piperidin-1-ium ring indicates that, overall, the mol­ecule has the shape of the letter *L*. This is confirmed by the +syn-clinal C3—C12—C13—C17 torsion angle of 60.1 (2)°.

In the anion, the r.m.s. deviation through the C_2_O_2_ atoms is 0.0131 Å with the Cl3 atom lying to one side of the plane [deviation = 1.7153 (3) Å] whereas the Cl1 [−0.9341 (3) Å] and Cl2 [−0.6170 (4) Å] atoms lie to the other side.

## Supra­molecular features   

**Figure 2:**
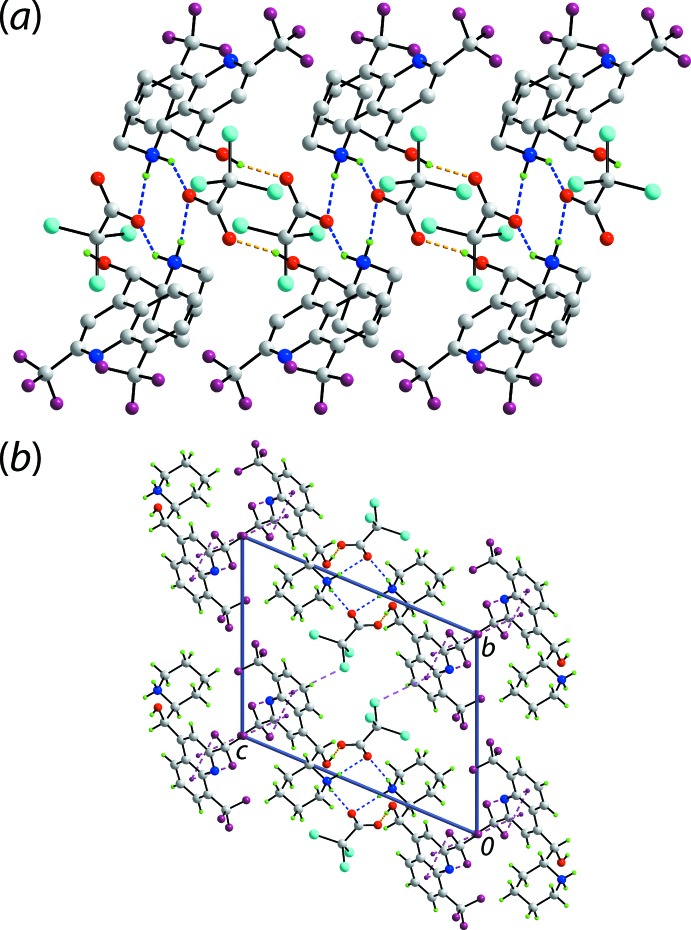
Mol­ecular packing in (I): (*a*) The supra­molecular chain along the *a* axis, being sustained by O—H⋯O and N—H⋯O hydrogen bonding with non-participating H atoms omitted and (*b*) a view of the unit-cell contents shown in projection down the *a* axis, the axis of propagation of the chain shown in (*a*). The C—Cl⋯π and C—F⋯π inter­actions are shown as pink and purple dashed lines, respectively.

The presence of charge-assisted hydrogen bonds between the constituent ions lead to linear, supra­molecular chains along the *a*-axis direction in the crystal of (I), Table 1 ▸ and Fig. 2 ▸(*a*). The most prominent feature of the packing is the formation of centrosymmetric, eight-membered {⋯O⋯HNH}_2_ synthons, which arise as a result of ammonium-N^+^—H⋯O^−^(carboxylate) hydrogen bonds whereby two ammonium cations bridge, via both hydrogen atoms, a pair of carboxyl­ate-O2 atoms. The four-ion aggregates are linked into the chain via charge-assisted hydroxyl-O—H⋯O^−^(carboxyl­ate) hydrogen bonds. These lead to larger centrosymmetric agglomerates, *i.e*. 18-membered {⋯OCO⋯HOC_2_NH}_2_ synthons. The connections between the chains are of the type C—*X*⋯π, for *X* = Cl and F. Such inter­actions are inherently weak, providing energies of stabilization less than 4 kcal mol^−1^, with those for inter­actions involving chloride atoms being greater than those with fluoride (Tsuzuki *et al.*, 2016 ▸). In the crystal of (I), C—Cl⋯π(C_6_-quinolin­yl) inter­actions are formed whereby the C—Cl bond is approximately parallel to the C_6_ ring. Each of the fluoride atoms bound to the C10 atom participates in a C—F⋯π contact as these CF_3_ groups lie in regions flanked by quinolinyl residues. Two of the contacts are as for the chloride atom, *i.e*. side on, whereas the other is best described as an end-on C—F⋯π contact as the angle subtended at the F1 atom is 170.95 (14)°. The aforementioned inter­actions combine to form a three-dimensional architecture. A view of the unit-cell contents is shown in Fig. 2 ▸(*b*).

## Hirshfeld surface analysis   

**Figure 3:**
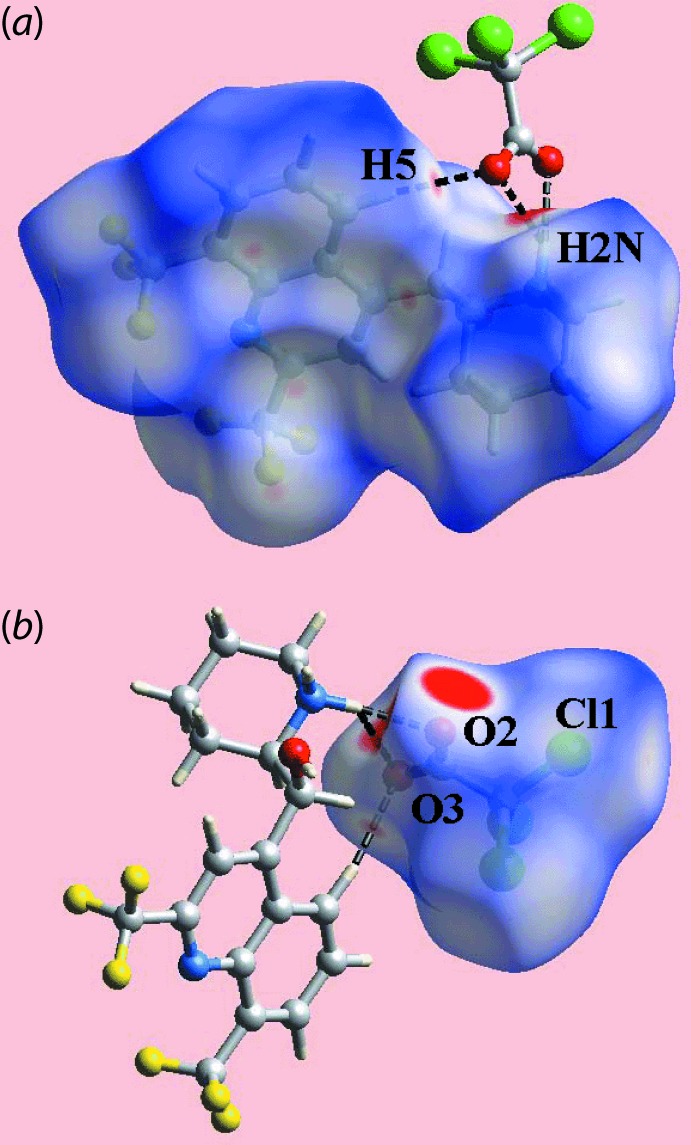
Views of the Hirshfeld surface of (I) mapped over *d*_norm_ in the range −0.171 to +1.475 a.u. for the (*a*) cation and (*b*) anion, highlighting N—H⋯O and C—H⋯O contacts as black dashed lines.

**Figure 4:**
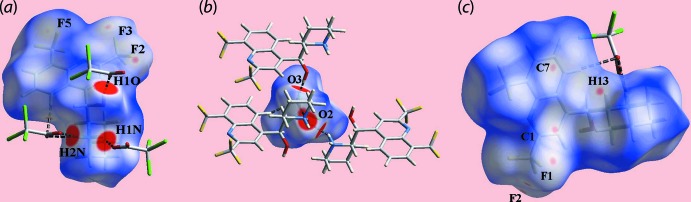
Views of the Hirshfeld surface of (I) mapped over *d*_norm_ in the range −0.121 to +1.475 a.u. for the (*a*) cation, (*b*) anion and (*c*) ion pair. The N—H⋯O, O—H⋯O and C—H⋯O contacts are shown as black dashed lines. The faint-red spots near the labelled atoms in (*b*) and (*c*) indicate the short inter­atomic contacts (see text and Table 2 ▸).

**Figure 5:**
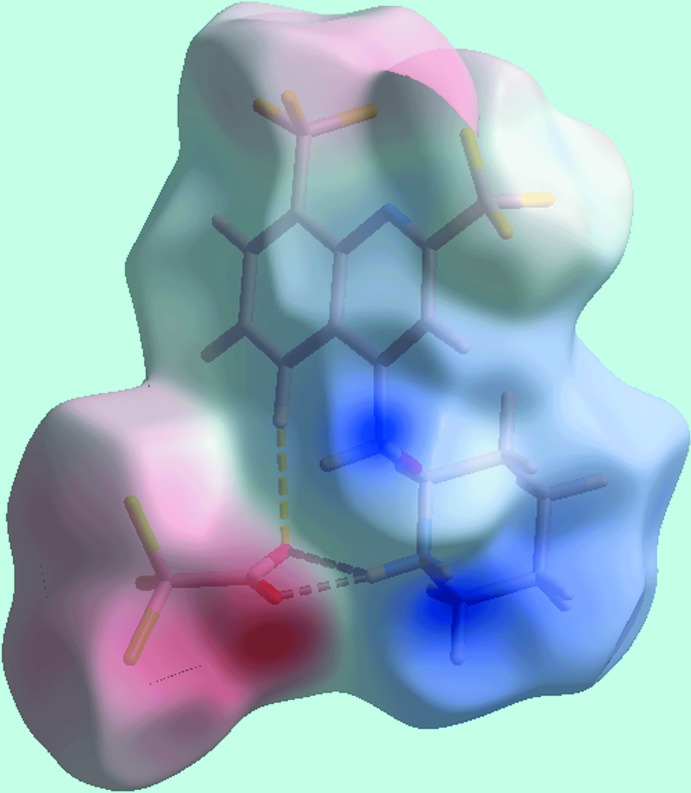
A view of the Hirshfeld surface of (I) mapped over the electrostatic potential in the range −0.128 to + 0.215 a.u. The red and blue regions represent negative and positive electrostatic potentials, respectively.

**Figure 6:**
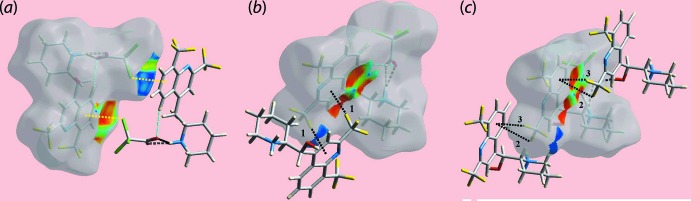
Three views of Hirshfeld surface of (I) mapped over the shape-index property highlighting (*a*) C—Cl⋯π/π⋯Cl—C contacts through yellow dotted lines, (*b*) and (*c*) C—F⋯π/π⋯F—C contacts with through black dotted lines. The ‘1′, ‘2′ and ‘3′ refer to the F1—F3 atoms, respectively

The Hirshfeld surface calculations for the title salt (I) were performed in accord with an earlier publication on a related organic salt (Jotani *et al.*, 2018 ▸). This analysis provides a convenient means to describe the formation of the salt through the charge-assisted N—H⋯O hydrogen bonds and C—H⋯O contacts, and the influence of weak inter­actions involving halide substituents in the crystal. The pair of overlapping bright-red spots near the ammonium-H2*N* atom and carboxyl­ate-O2 and O3 atoms of the anion on the Hirshfeld surfaces mapped over *d*_norm_ in Fig. 3 ▸ represent the charge-assisted N—H⋯O hydrogen-bonds; the methyl­ene-C13—H⋯O3 contact, Table 2 ▸, on the Hirshfeld surface is evident as the diminutive-red spot between the respective atoms in Fig. 3 ▸(*b*). The presence of bright- and broad-red spots near the ammonium-H1*N* and H2*N*, hydroxyl-H1*O*, carboxyl­ate-O2 and O3 atoms on the *d*_norm_-mapped Hirshfeld surfaces indicate the influence of the charge-assisted N—H⋯O and O—H⋯O hydrogen bonds, as indicated in Fig. 4 ▸(*a*) and (*b*). The donors and acceptors of inter­molecular inter­actions in the crystals of (I) are also highlighted with blue and red regions corresponding to positive and negative electrostatic potentials, respectively, on the Hirshfeld surfaces mapped over electrostatic potentials in Fig. 5 ▸. The presence of the faint-red spots near the CF_3_ atoms as well as the other atoms of the cation, Fig. 4 ▸(*a*) and (*c*), and the Cl1 atom, Fig. 3 ▸(*b*), indicate the involvement of these atoms in short inter­atomic contacts, Table 2 ▸. The effect of inter­molecular C—*X*⋯π inter­actions (*X* = F, Cl), Table 1 ▸, is illustrated in Fig. 6 ▸ through the blue and orange regions near the respective donors and acceptors on the Hirshfeld surfaces mapped with shape-index properties.

**Figure 7:**
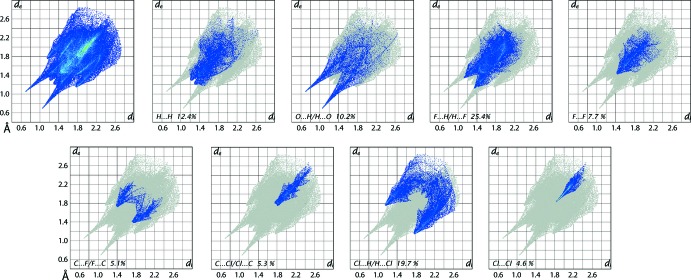
The full two-dimensional fingerprint plot for (I) and those delineated into H⋯H, O⋯H/H⋯O, F⋯H/H⋯F, F⋯F, C⋯F/F⋯C, C⋯Cl/Cl⋯C, Cl⋯H/H⋯Cl and Cl⋯Cl contacts.

The overall two-dimensional fingerprint plot for (I), Fig. 7 ▸, and those delineated into H⋯H, O⋯H/H⋯O, F⋯H/H⋯F, F⋯F, C⋯F/F⋯C, C⋯Cl/Cl⋯C, Cl⋯H/H⋯Cl and Cl⋯Cl contacts (McKinnon *et al.*, 2007 ▸) are illustrated in Fig. 7 ▸; the percentage contributions from the different inter­atomic contacts to the Hirshfeld surfaces are summarized in Table 3 ▸. The relatively small contribution, *i.e*. 12.4%, from H⋯H contacts to the Hirshfeld surfaces of (I) is due to the presence of terminal halide substituents in both the cation and anion and their relatively high contribution to a major portion of the surface.

The inter­molecular N—H⋯O and O—H⋯O hydrogen-bonds in the packing of (I) indicate a significant contribution from O⋯H/H⋯O contacts to the surface and these are evident as the two pairs of superimposed long spikes with the tips at *d*_e_ + *d*_i_ ∼1.7 Å in the delineated fingerprint plot. The largest percentage contribution to the Hirshfeld surface are from F⋯H/H⋯F contacts, *i.e*. 25.4%. This is due to the presence of a number of short inter­atomic H⋯F contacts, Table 2 ▸, which are characterized as the pair of short spikes at *d*_e_ + *d*_i_ ∼ 2.5 Å in the corresponding delineated fingerprint plot. An arrow-like tip at *d*_e_ + *d*_i_ ∼ 2.8 Å in the fingerprint delineated into F⋯F contacts is due to the effect of the short inter­atomic F⋯F contact summarized in Table 2 ▸. The presence of short inter­atomic C⋯F/F⋯C contacts, Table 2 ▸, and C—F⋯π contacts (Table 1 ▸) involving fluoride atoms substituted at the methyl-C10 atom is evident from the forceps-like distribution of points in the fingerprint plot delin­eated into these contacts. The C—Cl⋯π contact, Table 1 ▸, involving the carboxyl­ate-Cl3 atom, Fig. 6 ▸(*a*), is viewed as the spear-shaped distribution of points with the pair of adjoining tips at *d*_e_ + *d*_i_ ∼ 3.5 Å in the fingerprint plot delineated into C⋯Cl/Cl⋯C contacts. Although the inter­atomic Cl⋯H/H⋯Cl and Cl⋯Cl contacts make significant contributions to the Hirshfeld surface of (I), Table 3 ▸, and are reflected in the forceps-like and pencil-tip like distributions of points, respectively, in their delineated fingerprint plots, they occur at van der Waals separations. The small contribution from the other inter­atomic contacts to the Hirshfeld surface of (I), listed in Table 3 ▸, show negligible influence upon the packing.

## Database survey   

As noted in the *Chemical context*, there are two mefloquine derivatives that exhibit kryptoracemic behaviour with both examples being isolated after attempts at chiral resolution of racemic mefloquine with different carb­oxy­lic acids. In one example, two mefloquinium cations are related across a pseudo centre of inversion, and the charge balance is provided by two crystallographically independent 3,3,3-tri­fluoro-2-meth­oxy-2-phenyl­propano­ate anions, *i.e*. (+)-PhC(CF_3_)(OMe)CO_2_^−^ (Wardell, Wardell *et al.*, 2016 ▸). That it is not necessary to have chiral carboxyl­ate anions is seen in the second example of kryptoracemic behaviour whereby, as a result of incomplete substitution of chloride by 4-fluoro­benzene­sulfonate during an anion exchange experiment, the asymmetric unit comprises a pair of pseudo-enanti­omeric mefloquinium cations with equal numbers of chloride and 4-fluoro­benzene­sulfonate counter-ions (Jotani *et al.*, 2016 ▸).

There are a number of other structurally characterized mefloquinium salts, namely three isomeric *n*-nitro­benzoates (Wardell *et al.*, 2011 ▸), 3-amino-5-nitro­benzoate sesquihydrate (de Souza *et al.*, 2011 ▸), hy­droxy(phen­yl)acetate hemihydrate (Wardell, Jotani *et al.*, 2016 ▸) and tri­fluoro­acetate tri­fluoro­acetic acid hemihydrate (Low & Wardell, 2017 ▸), and all of these crystallize in centrosymmetric space groups with equal numbers of the mefloquinium enanti­omers. Further studies into the inter­esting phenomenon of kryptoracemic behaviour in mefloquinium salts are underway.

## Synthesis and crystallization   

A solution of mefloquinium chloride (1 mmol) and sodium di­fluoro­choro­acetate (1 mmol) in EtOH (10 ml) was refluxed for 20 min. The reaction mixture was left at room temperature and after two days, colourless slabs of (I) were collected; m.p. 473–475 K. ^1^H NMR (DMSO-*d*_6_) δ: 1.20–1.35(2H, *m*), 1.55–1.75(4H, *m*), 3.04 (1H, *br*,*t*), 3.53 (1H, *br.d*), 5.90 (1H, *s*), 6.94 (1H, *br.d*), 8.01 (1H, *t*, *J* = 8.0Hz), 8.13 (1H, *s*), 8.42 (1H, *d*, *J* = 8.02Hz), 8.72 (1H, *d*, *J* = 8.0Hz), 9.48 (1H, *br*,*s*); resonances due to O*H* and N*H* were not observed. ^13^C NMR (DMSO-d_6_) δ: 21.43 (2×), 21.59, 44.51, 58.90, 67.85, 135.50. 121.17 (*J*_C,F_ = 273.8 Hz), 121.21 (*J*_C,F_ = 311.0 Hz), 123.64 (*J*_C,F_ = 271.7 Hz), 126.37, 127.93 (*J*_C,F_ = 29.2 Hz), 128.32, 128.68. 129.9 (*J*_C,F_ = 5.2Hz), 142.78, 146.73 (*J*_C,F_ = 34.5 Hz), 150.97, 159.82 (*J*_C,F_ = 25.2 Hz). ^19^F NMR (DMSO-*d*_6_) δ: −58.65, −58.84, −66.68.

## Refinement   

Crystal data, data collection and structure refinement details are summarized in Table 4 ▸. The carbon-bound H atoms were placed in calculated positions (C—H = 0.95–1.00 Å) and were included in the refinement in the riding-model approximation, with *U*_iso_(H) set to 1.2*U*_eq_(C). The O- and N-bound H atoms were refined with distance restraints 0.84±0.01 and 0.88±0.01 Å, respectively, and refined with *U*_iso_(H) = 1.5*U*_eq_(O) and 1.2*U*_eq_(N), respectively. Owing to poor agreement, most likely due to inter­ference from the beam-stop, two reflections, *i.e*. (100) and (101), were omitted from the final cycles of refinement.

## Supplementary Material

Crystal structure: contains datablock(s) I, global. DOI: 10.1107/S2056989018016389/hb7786sup1.cif

Structure factors: contains datablock(s) I. DOI: 10.1107/S2056989018016389/hb7786Isup2.hkl

CCDC reference: 1879700

Additional supporting information:  crystallographic information; 3D view; checkCIF report

## Figures and Tables

**Table 1:** Hydrogen-bond geometry (Å, °) *Cg*1 and *Cg*2 are the centroids of the C4–C9 and N1/C1–C4/C9 rings, respectively.

*D*—H⋯*A*	*D*—H	H⋯*A*	*D*⋯*A*	*D*—H⋯*A*
N2—H1*N*⋯O1	0.88 (2)	2.42 (2)	2.734 (3)	102 (2)
N2—H1*N*⋯O2^i^	0.88 (2)	1.99 (2)	2.769 (3)	146 (2)
O1—H1*O*⋯O3^ii^	0.83 (2)	1.89 (2)	2.702 (2)	165 (2)
N2—H2*N*⋯O2	0.88 (2)	2.00 (2)	2.869 (2)	173 (2)
N2—H2*N*⋯O3	0.88 (2)	2.47 (2)	3.043 (3)	124 (1)
C19—Cl3⋯*Cg*1^iii^	1.78 (1)	3.61 (1)	4.709 (3)	118 (1)
C10—F1⋯*Cg*2^iv^	1.34 (1)	3.07 (1)	4.395 (2)	171 (1)
C10—F2⋯*Cg*1^ii^	1.34 (1)	3.44 (1)	3.788 (2)	95 (1)
C10—F3⋯*Cg*1^ii^	1.32 (1)	3.24 (1)	3.788 (2)	104 (1)

Symmetry codes: (i) 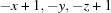; (ii) 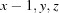; (iii) 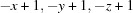; (iv) 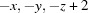.

**Table 2:** Summary of short inter­atomic contacts (Å) in (I)^*a*^

Contact	Distance	Symmetry operation
C1⋯F1	3.130 (2)	−*x*, −*y*, 2 − *z*
C7⋯F3	3.127 (3)	1 + *x*, *y*, *z*
O3⋯H13	2.49	*x*, *y*, *z*
F1⋯F2	2.8618 (18)	−1 − *x*, −*y*, 2 − *z*
F1⋯H17*B*	2.58	−*x*, −*y*, 2 − *z*
F5⋯Cl1	3.2065 (16)	1 − *x*, 1 − *y*, 1 − *z*
F2⋯H13	2.46	−1 + *x*, *y*, *z*

Note: (*a*) Values are as calculated in *CrystalExplorer* (Spackman & Jayatilaka, 2009 ▸).

**Table 3:** Percentage contributions of inter­atomic contacts to the Hirshfeld surface for (I)

	Percentage contribution
Contact	(I)
H⋯H	12.4
O⋯H/H⋯O	10.2
F⋯H/H⋯F	25.4
F⋯F	7.7
Cl⋯H/H⋯Cl	19.7
C⋯F/F⋯C	5.4
C⋯Cl/Cl⋯C	5.3
Cl⋯Cl	4.6
C⋯H/H⋯C	3.4
Cl⋯F/F⋯Cl	2.7
N⋯H/H⋯N	1.3
N⋯F/F⋯N	1.0
O⋯O	0.5
C⋯C	0.3
C⋯O/O⋯C	0.2
Cl⋯O/O⋯Cl	0.1

**Table 4:** Experimental details

Crystal data	
Chemical formula	C_17_H_17_F_6_N_2_O·C_2_Cl_3_O_2_
*M*_r_	541.69
Crystal system, space group	Triclinic, *P* 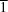
Temperature (K)	120
*a*, *b*, *c* (Å)	6.8087 (2), 11.8568 (5), 15.2562 (6)
α, β, γ (°)	67.473 (2), 81.663 (2), 89.824 (3)
*V* (Å^3^)	1123.77 (7)
*Z*	2
Radiation type	Mo *K*α
μ (mm^−1^)	0.48
Crystal size (mm)	0.30 × 0.26 × 0.21

Data collection	
Diffractometer	Bruker–Nonius Roper CCD camera on κ-goniostat
Absorption correction	Multi-scan (*SADABS*; Sheldrick, 2007 ▸)
*T*_min_, *T*_max_	0.636, 0.746
No. of measured, independent and observed [*I* > 2σ(*I*)] reflections	23628, 5114, 3880
*R*_int_	0.055
(sin θ/λ)_max_ (Å^−1^)	0.648

Refinement	
*R*[*F* ^2^ > 2σ(*F* ^2^)], *wR*(*F* ^2^), *S*	0.047, 0.133, 1.02
No. of reflections	5114
No. of parameters	307
No. of restraints	3
H-atom treatment	H atoms treated by a mixture of independent and constrained refinement
Δρ_max_, Δρ_min_ (e Å^−3^)	0.81, −0.59

Computer programs: *DENZO* (Otwinowski & Minor, 1997 ▸), *COLLECT* (Hooft, 1998 ▸), *SHELXS* (Sheldrick, 2008 ▸), *SHELXL2014/7* (Sheldrick, 2015 ▸), *ORTEP-3 for Windows* (Farrugia, 2012 ▸), *DIAMOND* (Brandenburg, 2006 ▸) and *publCIF* (Westrip, 2010 ▸).

## supplementary crystallographic information

### Crystal data

**Table:**

C_17_H_17_F_6_N_2_O·C_2_Cl_3_O_2_	*Z* = 2
*M**_r_* = 541.69	*F*(000) = 548
Triclinic, *P*1	*D*_x_ = 1.601 Mg m^−^^3^
*a* = 6.8087 (2) Å	Mo *K*α radiation, λ = 0.71073 Å
*b* = 11.8568 (5) Å	Cell parameters from 25711 reflections
*c* = 15.2562 (6) Å	θ = 2.9–27.5°
α = 67.473 (2)°	µ = 0.48 mm^−^^1^
β = 81.663 (2)°	*T* = 120 K
γ = 89.824 (3)°	Slab, colourless
*V* = 1123.77 (7) Å^3^	0.30 × 0.26 × 0.21 mm

### Data collection

**Table:**

Bruker–Nonius Roper CCD camera on κ-goniostat diffractometer	5114 independent reflections
Radiation source: Bruker-Nonius FR591 rotating anode	3880 reflections with *I* > 2σ(*I*)
Graphite monochromator	*R*_int_ = 0.055
Detector resolution: 9.091 pixels mm^-1^	θ_max_ = 27.4°, θ_min_ = 2.9°
φ & ω scans	*h* = −8→8
Absorption correction: multi-scan (*SADABS*; Sheldrick, 2007)	*k* = −15→15
*T*_min_ = 0.636, *T*_max_ = 0.746	*l* = −19→19
23628 measured reflections	

### Refinement

**Table:**

Refinement on *F*^2^	Primary atom site location: structure-invariant direct methods
Least-squares matrix: full	Hydrogen site location: mixed
*R*[*F*^2^ > 2σ(*F*^2^)] = 0.047	H atoms treated by a mixture of independent and constrained refinement
*wR*(*F*^2^) = 0.133	*w* = 1/[σ^2^(*F*_o_^2^) + (0.0695*P*)^2^ + 0.5906*P*] where *P* = (*F*_o_^2^ + 2*F*_c_^2^)/3
*S* = 1.02	(Δ/σ)_max_ < 0.001
5114 reflections	Δρ_max_ = 0.81 e Å^−^^3^
307 parameters	Δρ_min_ = −0.59 e Å^−^^3^
3 restraints	

### Special details

**Table:**

Geometry. All esds (except the esd in the dihedral angle between two l.s. planes) are estimated using the full covariance matrix. The cell esds are taken into account individually in the estimation of esds in distances, angles and torsion angles; correlations between esds in cell parameters are only used when they are defined by crystal symmetry. An approximate (isotropic) treatment of cell esds is used for estimating esds involving l.s. planes.
Refinement. Owing to poor agreement, perhaps owing to interference from the beam-stop, two reflections, i.e. (1 0 0) and (1 0 1), were omitted from the final cycles of the refinement.

### Fractional atomic coordinates and isotropic or equivalent isotropic displacement parameters (Å^2^)

**Table:**

	*x*	*y*	*z*	*U*_iso_*/*U*_eq_	
F1	−0.22880 (19)	−0.00170 (13)	1.00487 (9)	0.0378 (3)	
F2	−0.38289 (18)	0.05158 (14)	0.88298 (9)	0.0392 (3)	
F3	−0.36487 (19)	0.17017 (13)	0.95862 (9)	0.0384 (3)	
F4	0.1664 (2)	0.31406 (13)	1.01713 (9)	0.0430 (3)	
F5	0.0401 (2)	0.46009 (13)	0.91079 (10)	0.0454 (4)	
F6	0.3286 (3)	0.48695 (15)	0.94377 (13)	0.0590 (5)	
O1	0.1401 (2)	0.03503 (14)	0.64074 (11)	0.0300 (3)	
H1O	0.058 (3)	0.084 (2)	0.6145 (19)	0.045*	
N1	0.0200 (3)	0.23331 (16)	0.88257 (12)	0.0255 (4)	
N2	0.5142 (3)	−0.04940 (16)	0.63900 (12)	0.0246 (4)	
H1N	0.420 (3)	−0.084 (2)	0.6219 (17)	0.029*	
H2N	0.565 (3)	0.0117 (16)	0.5858 (11)	0.029*	
C1	−0.0699 (3)	0.15108 (19)	0.86236 (14)	0.0248 (4)	
C2	0.0070 (3)	0.10584 (19)	0.79255 (14)	0.0253 (4)	
H2	−0.0687	0.0471	0.7811	0.030*	
C3	0.1930 (3)	0.14755 (18)	0.74109 (14)	0.0232 (4)	
C4	0.2993 (3)	0.23707 (18)	0.76020 (14)	0.0234 (4)	
C5	0.4941 (3)	0.28638 (19)	0.71304 (15)	0.0280 (4)	
H5	0.5585	0.2614	0.6645	0.034*	
C6	0.5892 (3)	0.3690 (2)	0.73676 (17)	0.0330 (5)	
H6	0.7195	0.4006	0.7048	0.040*	
C7	0.4967 (4)	0.4085 (2)	0.80829 (17)	0.0341 (5)	
H7	0.5657	0.4656	0.8244	0.041*	
C8	0.3084 (3)	0.36492 (19)	0.85441 (15)	0.0294 (5)	
C9	0.2044 (3)	0.27756 (18)	0.83188 (14)	0.0244 (4)	
C10	−0.2631 (3)	0.0942 (2)	0.92713 (15)	0.0283 (4)	
C11	0.2098 (4)	0.4061 (2)	0.93101 (17)	0.0365 (5)	
C12	0.2837 (3)	0.09461 (19)	0.66926 (14)	0.0236 (4)	
H12	0.3575	0.1615	0.6113	0.028*	
C13	0.4279 (3)	−0.00337 (18)	0.71443 (14)	0.0233 (4)	
H13	0.5380	0.0361	0.7315	0.028*	
C14	0.6690 (3)	−0.1405 (2)	0.67050 (17)	0.0317 (5)	
H14A	0.7833	−0.1014	0.6836	0.038*	
H14B	0.7175	−0.1693	0.6187	0.038*	
C15	0.5805 (3)	−0.2485 (2)	0.76066 (17)	0.0330 (5)	
H15A	0.6859	−0.3051	0.7841	0.040*	
H15B	0.4774	−0.2935	0.7452	0.040*	
C16	0.4882 (4)	−0.2063 (2)	0.83963 (16)	0.0332 (5)	
H16A	0.4233	−0.2776	0.8957	0.040*	
H16B	0.5940	−0.1701	0.8607	0.040*	
C17	0.3350 (3)	−0.11181 (19)	0.80364 (15)	0.0291 (5)	
H17A	0.2230	−0.1503	0.7883	0.035*	
H17B	0.2819	−0.0829	0.8548	0.035*	
Cl1	0.91440 (9)	0.34265 (7)	0.30595 (4)	0.04821 (19)	
Cl2	1.10599 (11)	0.35855 (7)	0.45615 (5)	0.0571 (2)	
Cl3	0.70646 (12)	0.44205 (6)	0.43618 (6)	0.0577 (2)	
O2	0.6471 (2)	0.16019 (15)	0.46652 (12)	0.0369 (4)	
O3	0.8236 (2)	0.16197 (15)	0.57789 (11)	0.0352 (4)	
C18	0.7750 (3)	0.20401 (19)	0.49632 (15)	0.0259 (4)	
C19	0.8759 (3)	0.3309 (2)	0.42622 (16)	0.0319 (5)	

### Atomic displacement parameters (Å^2^)

**Table:**

	*U*^11^	*U*^22^	*U*^33^	*U*^12^	*U*^13^	*U*^23^
F1	0.0324 (7)	0.0401 (8)	0.0286 (7)	0.0043 (6)	−0.0029 (5)	−0.0006 (6)
F2	0.0299 (7)	0.0574 (9)	0.0320 (7)	−0.0063 (6)	−0.0034 (5)	−0.0194 (7)
F3	0.0352 (7)	0.0435 (8)	0.0343 (7)	0.0093 (6)	0.0029 (6)	−0.0156 (6)
F4	0.0635 (9)	0.0430 (8)	0.0268 (7)	0.0074 (7)	−0.0131 (6)	−0.0160 (6)
F5	0.0657 (10)	0.0403 (8)	0.0359 (8)	0.0224 (7)	−0.0090 (7)	−0.0208 (7)
F6	0.0770 (11)	0.0524 (10)	0.0639 (11)	−0.0129 (8)	−0.0020 (9)	−0.0435 (9)
O1	0.0328 (8)	0.0348 (9)	0.0298 (8)	0.0081 (6)	−0.0138 (6)	−0.0174 (7)
N1	0.0295 (9)	0.0247 (9)	0.0230 (8)	0.0073 (7)	−0.0070 (7)	−0.0088 (7)
N2	0.0304 (9)	0.0238 (9)	0.0227 (9)	0.0022 (7)	−0.0058 (7)	−0.0118 (7)
C1	0.0274 (10)	0.0259 (10)	0.0204 (10)	0.0069 (8)	−0.0062 (8)	−0.0072 (8)
C2	0.0281 (10)	0.0247 (10)	0.0243 (10)	0.0032 (8)	−0.0076 (8)	−0.0097 (8)
C3	0.0292 (10)	0.0202 (10)	0.0200 (9)	0.0044 (8)	−0.0059 (8)	−0.0068 (8)
C4	0.0290 (10)	0.0187 (9)	0.0216 (10)	0.0052 (8)	−0.0066 (8)	−0.0060 (8)
C5	0.0324 (11)	0.0231 (10)	0.0258 (10)	0.0023 (8)	−0.0023 (8)	−0.0073 (8)
C6	0.0346 (11)	0.0231 (11)	0.0356 (12)	−0.0030 (9)	−0.0024 (9)	−0.0062 (9)
C7	0.0433 (13)	0.0210 (10)	0.0381 (13)	−0.0012 (9)	−0.0123 (10)	−0.0092 (9)
C8	0.0416 (12)	0.0208 (10)	0.0269 (11)	0.0042 (9)	−0.0084 (9)	−0.0094 (9)
C9	0.0310 (10)	0.0203 (10)	0.0220 (10)	0.0052 (8)	−0.0073 (8)	−0.0072 (8)
C10	0.0301 (11)	0.0312 (11)	0.0239 (10)	0.0066 (9)	−0.0068 (8)	−0.0103 (9)
C11	0.0532 (14)	0.0278 (11)	0.0333 (12)	0.0029 (10)	−0.0092 (11)	−0.0162 (10)
C12	0.0274 (10)	0.0253 (10)	0.0195 (9)	0.0032 (8)	−0.0056 (8)	−0.0094 (8)
C13	0.0284 (10)	0.0240 (10)	0.0206 (9)	0.0032 (8)	−0.0050 (8)	−0.0117 (8)
C14	0.0305 (11)	0.0312 (12)	0.0374 (12)	0.0072 (9)	−0.0062 (9)	−0.0174 (10)
C15	0.0360 (12)	0.0257 (11)	0.0382 (13)	0.0059 (9)	−0.0094 (10)	−0.0121 (10)
C16	0.0425 (12)	0.0259 (11)	0.0289 (11)	0.0054 (9)	−0.0102 (9)	−0.0063 (9)
C17	0.0361 (11)	0.0272 (11)	0.0225 (10)	0.0056 (9)	−0.0053 (9)	−0.0077 (9)
Cl1	0.0439 (3)	0.0694 (5)	0.0241 (3)	0.0045 (3)	−0.0020 (2)	−0.0114 (3)
Cl2	0.0573 (4)	0.0519 (4)	0.0510 (4)	−0.0245 (3)	−0.0205 (3)	−0.0033 (3)
Cl3	0.0819 (5)	0.0298 (3)	0.0581 (4)	0.0197 (3)	−0.0082 (4)	−0.0145 (3)
O2	0.0414 (9)	0.0423 (9)	0.0345 (9)	−0.0046 (7)	−0.0039 (7)	−0.0238 (8)
O3	0.0335 (8)	0.0359 (9)	0.0297 (8)	0.0021 (7)	−0.0083 (7)	−0.0045 (7)
C18	0.0260 (10)	0.0250 (10)	0.0294 (11)	0.0046 (8)	−0.0019 (8)	−0.0145 (9)
C19	0.0379 (12)	0.0302 (12)	0.0265 (11)	0.0027 (9)	−0.0079 (9)	−0.0088 (9)

### Geometric parameters (Å, º)

**Table:**

F1—C10	1.341 (2)	C7—C8	1.367 (3)
F2—C10	1.341 (2)	C7—H7	0.9500
F3—C10	1.324 (2)	C8—C9	1.429 (3)
F4—C11	1.340 (3)	C8—C11	1.504 (3)
F5—C11	1.337 (3)	C12—C13	1.538 (3)
F6—C11	1.344 (3)	C12—H12	1.0000
O1—C12	1.417 (2)	C13—C17	1.523 (3)
O1—H1O	0.835 (10)	C13—H13	1.0000
N1—C1	1.307 (3)	C14—C15	1.519 (3)
N1—C9	1.364 (3)	C14—H14A	0.9900
N2—C13	1.500 (2)	C14—H14B	0.9900
N2—C14	1.501 (3)	C15—C16	1.529 (3)
N2—H1N	0.882 (10)	C15—H15A	0.9900
N2—H2N	0.879 (10)	C15—H15B	0.9900
C1—C2	1.404 (3)	C16—C17	1.527 (3)
C1—C10	1.510 (3)	C16—H16A	0.9900
C2—C3	1.372 (3)	C16—H16B	0.9900
C2—H2	0.9500	C17—H17A	0.9900
C3—C4	1.428 (3)	C17—H17B	0.9900
C3—C12	1.518 (3)	Cl1—C19	1.766 (2)
C4—C9	1.428 (3)	Cl2—C19	1.759 (2)
C4—C5	1.423 (3)	Cl3—C19	1.784 (2)
C5—C6	1.361 (3)	O2—C18	1.238 (3)
C5—H5	0.9500	O3—C18	1.245 (3)
C6—C7	1.413 (3)	C18—C19	1.562 (3)
C6—H6	0.9500		
			
C12—O1—H1O	110 (2)	F6—C11—C8	111.3 (2)
C1—N1—C9	116.91 (17)	O1—C12—C3	112.85 (16)
C13—N2—C14	113.36 (16)	O1—C12—C13	105.73 (16)
C13—N2—H1N	110.4 (15)	C3—C12—C13	110.13 (16)
C14—N2—H1N	108.4 (16)	O1—C12—H12	109.3
C13—N2—H2N	110.7 (16)	C3—C12—H12	109.3
C14—N2—H2N	109.4 (16)	C13—C12—H12	109.3
H1N—N2—H2N	104 (2)	N2—C13—C17	108.72 (16)
N1—C1—C2	125.37 (19)	N2—C13—C12	107.06 (16)
N1—C1—C10	115.36 (18)	C17—C13—C12	115.11 (17)
C2—C1—C10	119.01 (18)	N2—C13—H13	108.6
C3—C2—C1	119.12 (19)	C17—C13—H13	108.6
C3—C2—H2	120.4	C12—C13—H13	108.6
C1—C2—H2	120.4	N2—C14—C15	109.75 (17)
C2—C3—C4	118.17 (18)	N2—C14—H14A	109.7
C2—C3—C12	119.94 (18)	C15—C14—H14A	109.7
C4—C3—C12	121.84 (17)	N2—C14—H14B	109.7
C9—C4—C5	118.59 (18)	C15—C14—H14B	109.7
C9—C4—C3	117.62 (18)	H14A—C14—H14B	108.2
C5—C4—C3	123.78 (18)	C14—C15—C16	111.18 (18)
C6—C5—C4	120.7 (2)	C14—C15—H15A	109.4
C6—C5—H5	119.6	C16—C15—H15A	109.4
C4—C5—H5	119.6	C14—C15—H15B	109.4
C5—C6—C7	121.0 (2)	C16—C15—H15B	109.4
C5—C6—H6	119.5	H15A—C15—H15B	108.0
C7—C6—H6	119.5	C17—C16—C15	110.84 (18)
C8—C7—C6	120.3 (2)	C17—C16—H16A	109.5
C8—C7—H7	119.9	C15—C16—H16A	109.5
C6—C7—H7	119.9	C17—C16—H16B	109.5
C7—C8—C9	120.4 (2)	C15—C16—H16B	109.5
C7—C8—C11	120.5 (2)	H16A—C16—H16B	108.1
C9—C8—C11	119.1 (2)	C13—C17—C16	110.83 (18)
N1—C9—C4	122.80 (18)	C13—C17—H17A	109.5
N1—C9—C8	118.09 (18)	C16—C17—H17A	109.5
C4—C9—C8	119.07 (19)	C13—C17—H17B	109.5
F3—C10—F1	106.88 (17)	C16—C17—H17B	109.5
F3—C10—F2	107.34 (17)	H17A—C17—H17B	108.1
F1—C10—F2	106.53 (17)	O2—C18—O3	127.3 (2)
F3—C10—C1	113.66 (18)	O2—C18—C19	116.35 (19)
F1—C10—C1	110.42 (16)	O3—C18—C19	116.19 (18)
F2—C10—C1	111.64 (17)	C18—C19—Cl2	111.80 (15)
F5—C11—F4	106.7 (2)	C18—C19—Cl1	111.57 (15)
F5—C11—F6	106.71 (19)	Cl2—C19—Cl1	108.50 (12)
F4—C11—F6	105.65 (19)	C18—C19—Cl3	105.96 (14)
F5—C11—C8	113.08 (19)	Cl2—C19—Cl3	110.39 (13)
F4—C11—C8	112.85 (19)	Cl1—C19—Cl3	108.56 (12)
			
C9—N1—C1—C2	−0.6 (3)	N1—C1—C10—F2	154.97 (17)
C9—N1—C1—C10	173.54 (17)	C2—C1—C10—F2	−30.5 (3)
N1—C1—C2—C3	1.3 (3)	C7—C8—C11—F5	120.8 (2)
C10—C1—C2—C3	−172.61 (18)	C9—C8—C11—F5	−60.6 (3)
C1—C2—C3—C4	−0.9 (3)	C7—C8—C11—F4	−118.0 (2)
C1—C2—C3—C12	176.57 (18)	C9—C8—C11—F4	60.7 (3)
C2—C3—C4—C9	−0.1 (3)	C7—C8—C11—F6	0.7 (3)
C12—C3—C4—C9	−177.48 (17)	C9—C8—C11—F6	179.31 (19)
C2—C3—C4—C5	179.01 (19)	C2—C3—C12—O1	19.3 (3)
C12—C3—C4—C5	1.6 (3)	C4—C3—C12—O1	−163.38 (17)
C9—C4—C5—C6	1.0 (3)	C2—C3—C12—C13	−98.6 (2)
C3—C4—C5—C6	−178.1 (2)	C4—C3—C12—C13	78.7 (2)
C4—C5—C6—C7	−0.4 (3)	C14—N2—C13—C17	−58.8 (2)
C5—C6—C7—C8	−0.6 (3)	C14—N2—C13—C12	176.26 (16)
C6—C7—C8—C9	1.0 (3)	O1—C12—C13—N2	58.90 (19)
C6—C7—C8—C11	179.6 (2)	C3—C12—C13—N2	−178.90 (15)
C1—N1—C9—C4	−0.5 (3)	O1—C12—C13—C17	−62.1 (2)
C1—N1—C9—C8	−178.23 (18)	C3—C12—C13—C17	60.1 (2)
C5—C4—C9—N1	−178.32 (18)	C13—N2—C14—C15	57.8 (2)
C3—C4—C9—N1	0.8 (3)	N2—C14—C15—C16	−54.6 (2)
C5—C4—C9—C8	−0.6 (3)	C14—C15—C16—C17	55.1 (2)
C3—C4—C9—C8	178.53 (18)	N2—C13—C17—C16	57.1 (2)
C7—C8—C9—N1	177.45 (19)	C12—C13—C17—C16	177.17 (17)
C11—C8—C9—N1	−1.2 (3)	C15—C16—C17—C13	−56.5 (2)
C7—C8—C9—C4	−0.4 (3)	O2—C18—C19—Cl2	−159.24 (16)
C11—C8—C9—C4	−179.02 (19)	O3—C18—C19—Cl2	25.0 (2)
N1—C1—C10—F3	33.4 (2)	O2—C18—C19—Cl1	−37.5 (2)
C2—C1—C10—F3	−152.12 (18)	O3—C18—C19—Cl1	146.68 (16)
N1—C1—C10—F1	−86.7 (2)	O2—C18—C19—Cl3	80.4 (2)
C2—C1—C10—F1	87.8 (2)	O3—C18—C19—Cl3	−95.35 (19)

### Hydrogen-bond geometry (Å, º)

Cg1 and Cg2 are the centroids of the C4–C9 and N1/C1–C4/C9 rings, respectively.

**Table:**

*D*—H···*A*	*D*—H	H···*A*	*D*···*A*	*D*—H···*A*
N2—H1*N*···O1	0.88 (2)	2.42 (2)	2.734 (3)	102 (2)
N2—H1*N*···O2^i^	0.88 (2)	1.99 (2)	2.769 (3)	146 (2)
O1—H1*O*···O3^ii^	0.83 (2)	1.89 (2)	2.702 (2)	165 (2)
N2—H2*N*···O2	0.88 (2)	2.00 (2)	2.869 (2)	173 (2)
N2—H2*N*···O3	0.88 (2)	2.47 (2)	3.043 (3)	124 (1)
C19—Cl3···*Cg*1^iii^	1.78 (1)	3.61 (1)	4.709 (3)	118 (1)
C10—F1···*Cg*2^iv^	1.34 (1)	3.07 (1)	4.395 (2)	171 (1)
C10—F2···*Cg*1^ii^	1.34 (1)	3.44 (1)	3.788 (2)	95 (1)
C10—F3···*Cg*1^ii^	1.32 (1)	3.24 (1)	3.788 (2)	104 (1)

Symmetry codes: (i) −*x*+1, −*y*, −*z*+1; (ii) *x*−1, *y*, *z*; (iii) −*x*+1, −*y*+1, −*z*+1; (iv) −*x*, −*y*, −*z*+2.
